# FTY720/Fingolimod, a Sphingosine Analogue, Reduces Amyloid-β Production in Neurons

**DOI:** 10.1371/journal.pone.0064050

**Published:** 2013-05-07

**Authors:** Nobumasa Takasugi, Tomoki Sasaki, Ihori Ebinuma, Satoko Osawa, Hayato Isshiki, Koji Takeo, Taisuke Tomita, Takeshi Iwatsubo

**Affiliations:** 1 Department of Neuropathology and Neuroscience, Graduate School of Pharmaceutical Sciences, The University of Tokyo, Tokyo, Japan; 2 Department of Neuropathology, Graduate School of Medicine, The University of Tokyo, Tokyo, Japan; 3 Core Research for Evolutional Science and Technology, Japan Science and Technology Agency, The University of Tokyo, Tokyo, Japan; Thomas Jefferson University, United States of America

## Abstract

Sphingosine-1-phosphate (S1P) is a pluripotent lipophilic mediator working as a ligand for G-protein coupled S1P receptors (S1PR), which is currently highlighted as a therapeutic target for autoimmune diseases including relapsing forms of multiple sclerosis. Sphingosine related compounds, FTY720 and KRP203 known as S1PR modulators, are phosphorylated by sphingosine kinase 2 (SphK2) to yield the active metabolites FTY720-P and KRP203-P, which work as functional antagonists for S1PRs. Here we report that FTY720 and KRP203 decreased production of Amyloid-β peptide (Aβ), a pathogenic proteins causative for Alzheimer disease (AD), in cultured neuronal cells. Pharmacological analyses suggested that the mechanism of FTY720-mediated Aβ decrease in cells was independent of known downstream signaling pathways of S1PRs. Unexpectedly, 6-days treatment of APP transgenic mice with FTY720 resulted in a decrease in Aβ40, but an increase in Aβ42 levels in brains. These results suggest that S1PR modulators are novel type of regulators for Aβ metabolisms that are active *in vitro* and *in vivo*.

## Introduction

Bioactive lipids, such as sphingolipids, have effect on various neuronal activities, including signal transduction, inflammatory response, and neuronal survivals [1]. It has been reported that the sphingolipid metabolism in brain was altered under neurodegenerative conditions, e.g., Alzheimer disease (AD) [2], [3], [4]. However, the relationship between changes in brain sphingolipids with the pathological mechanisms of AD has remained largely unclear. Interestingly, the production of amyloid-β (Aβ) peptide, the major component of senile plaques deposited in the brains of patients with AD, is known to be modulated by sphingolipids [5]. Aβ is produced from amyloid-β precursor protein (APP) through a sequential cleavage by two aspartate proteases, β- and γ-secretases [6], [7]. BACE1 (β-site APP cleaving enzyme 1) [8] is a type-1 transmembrane protein responsible for the β-secretase activity, and γ-secretase is comprised of four integral membrane proteins, Presenilin (PS) as the catalytic subunit associated with Nicastrin (Nct), Aph1, and Pen2 [9]. Both enzymes are located in lipid rafts [10], a membrane microdomain enriched in sphingolipids and cholesterol, and the activities of the secretases are affected by the lipid composition [11], [12], [13].

**Figure 1:**
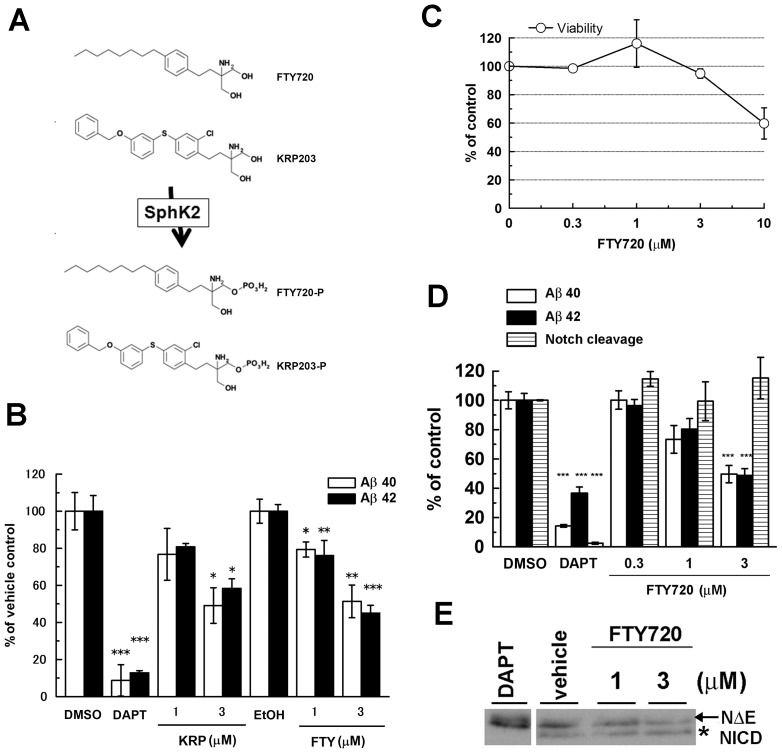
S1P receptor modulators, FTY720 and KRP203 decreased Aβ production from neuronal cells. (**A**) The chemical structures of FTY720 and KRP203 and their phosphorylated forms. (**B**) Levels of Aβ secretion from mouse primary neurons after treatment with FTY720 or KRP203 for 24 hrs. The levels of secreted Aβ in conditioned media were quantified by ELISAs. For vehicle control, we used DMSO for FTY720 treatment and EtOH for KRP203 treatment, respectively. The percentages of the relative ratio to levels in vehicle control of each group (mean ± SEM) are indicated in the figures. *P<0.05, ***P<0.001 by Student's t test. (n = 4). (**C**) Effects of FTY720 on cell viability in N2aNH cells. (**D**) Levels of Aβ secretion and Notch activity in N2aNH cells after treatment with FTY720 (n = 4, mean ± SEM; *P<0.05, **P<0.01, ***P<0.001). 10 µM of DAPT was used as a positive control. (**E**) Immunoblot analysis of NICD in FTY720-treated N2a cells, which was transiently transfected with cDNA encoding NΔE.

Sphingosine-1-phosphate (S1P) is produced from sphingosine by sphingosine kinase (SphK). S1P works as a ligand for a subset of G-protein coupled receptor (S1PR) proteins, and functions on various cellular events including neurogenesis, angiogenesis, and immune response [14]. S1PR modulator FTY720 (Fingolimod/Gilenya) is a sphingosine-related molecule exhibiting an immunomodulatory function, which has recently been approved as an oral treatment for relapsing forms of multiple sclerosis [14], [15]. FTY720 is phosphorylated by SphK2 to function as an agonist for S1P receptors, i.e., S1PR1, S1PR3, S1PR4, and S1PR5 (Fig. 1) [16], [17], [18]. Despite its agonistic action, FTY720 promotes endocytosis and degradation of the S1P receptors, thereby resulting in functional antagonistic effects. FTY720 interferes with the neuroinflammatory responses of auto-active T-cells and glial cells [19] and ameliorates the symptoms of autoimmune encephalomyelitis in rodents, the latter being a model for multiple sclerosis [20], [21].

Recently, we reported that SphK2, one of the rate limiting enzymes for the production of S1P, is upregulated in AD brains, and that S1P interacts with BACE1 to regulate its proteolytic activity [22]. To examine the effects of S1P receptor modulators on AD, we investigated the effects of FTY720, and another S1PR modulator KRP203 harboring a more specific agonist activity against S1PR1 [23], on Aβ production in cultured neuronal cells and brain Aβ levels in AD model mice.

## Materials and Methods

### Compounds

2-amino-2-[2-(4-octylphenyl)ethyl]-1,3-propanediol, hydrochloride (FTY720), 2-amino-2-[2-[2-chloro-4-[[3-(phenylmethoxy)phenyl]thio]phenyl]ethyl]-1,3-propanediol, hydrochloride (KRP203), 3-(2-(3-hexylphenylamino)-2-oxoethylamino) propanoic acid (W123), 5-[4-phenyl-5-(trifluoromethyl)-2-thienyl]-3-[3-(trifluoromethyl)phenyl]-1,2,4-oxadiazole (SEW2871) (Cayman chemicals), phosphorylated form of FTY720 (FTY720-P) (Echelon bioscience), and suramin (Sigma-Aldrich) were purchased from indicated venders. *N*-[*N*-(3,5-difluorophenacetyl)-L-alanyl]-(*S*)-phenylglycine *t*-butyl ester (DAPT) was synthesized as previously described [24]. FTY720, W123, suramin and DAPT were dissolved in DMSO. KRP203 and FTY720-P was dissolved in ethanol and chloroform, respectively.

### Antibodies and immunological methods

A polyclonal antibody against presnilin1 (PS1) CTF (G1L3) was raised as described [25]. Following antibodies were purchased from indicated venders: 82E1 (Immuno-Biological Laboratories) against the N terminus of human Aβ for detection of Aβ and βCTF, anti-APP (C) (Immuno-Biological Laboratories) for detection of APP CTFs and AICD, anti-mouse/rat APP (597) for detection of sAPPα, anti-sAPPβwt (Immuno-Biological Laboratories), anti-α-Tubulin DM1A (Sigma Aldrich), anti-SphK2 P-19 (Santa Cruz Biotechnology), anti-Myc 9B11 and anti-phospho specific ERK antibody (Cell Signaling Technology). Protein samples were analyzed by immunoblotting or two-site ELISAs for the detection of Aβ as previously described [26], [27]. Specificities of the APP antibodies used have previously been shown [22]. To analyze Aβ species with different C-terminal lengths, samples were separated by modified Tris/Tricine/8M urea gels as reported previously, followed by immunoblotting with 82E1 [28], [29].

### Cell culture and transfection

Expression plasmids coding for human APP C-terminal 99 amino acid fragment (pcDNA3.1-SC100), Notch C-terminal fragment (pCS2-NΔE), human Sphingosine kinase 2 (pcDNA3.1-SphK2-V5) and an inactive mutant of SphK2 (G243D) were described previously [22], [26], [30], [31]. Plasmid transfection was performed using Lipofectamine2000 (Invitrogen). Stable mouse Neuro2a neuroblastoma cells line expressing recombinant Notch protein and luciferase reporter (N2aNH) was established by co-transfection with pcDNA3.1/Hygro-NΔE/gvp, pcDNA3-EGFP and pGL3(r2.2)-UAS [32], [33] followed by selection with G418 and hygromycin. After indicated time of treatment, medium and lysate were collected. To monitor the cell viability, we compared GFP fluorescence in each lysate with that of vehicle control samples. We further validated this viability assay using almarBlue assay (Invitrogen) (Figure S1). To monitor the Notch cleavage, luciferase assay was performed as described previously [29], [32], [33]. Primary cortical neurons were prepared from Balb/c mice at embryonic day 16, and grown in Neurobasal medium supplemented with B27 (Invitrogen) for 7 days [34], [35]. Small interfering RNA (siRNA) duplexes targeting the mouse *Sphk2* sequence (target sequences: *Sphk2*: 5′-TAG GCC TGG CCT CGT TGC ATA-3′) as well as a negative control sequence were purchased from Qiagen. siRNA was reversely transfected in N2a cells using Lipofectamine RNAiMax (Invitrogen) as previously described [22]. cDNAs encoding NΔE [30] and NΔEgv [36] were originally provided from Drs. Raphael Kopan and Jan Naslund, respectively.

### FTY720 treatment in AD model mice

All experiments using animals in this study were performed according to the guidelines provided by the Institutional Animal Care Committee of Graduate School of Pharmaceutical Sciences, The University of Tokyo. All animals were maintained on food and water with a 12 h light/dark cycle. A7 transgenic mice overexpressing human APP695 harboring K670N, M671L, and T714I FAD mutations in neurons under the control of Thy1.2 promoter were used as a mouse model of AD [37]. Female A7 mice at 6 months old were used for treatment of FTY720, in which FTY720 dissolved in saline was injected subcutaneously once a day for 6 days (0.5 mg/kg/day). Brain samples were solubilized with 10 mM Tris buffer containing 1% CHAPS, and subjected to the sandwich ELISA for Aβ (Wako Chemical) [22].

## Results

### S1P receptor modulators, FTY720 and KRP203, decreased Aβ production in neuronal cells

To test if the S1PR modulators alter Aβ production in neuronal cells, we treated mouse primary cortical neurons with FTY720 and KRP203 (structures shown in Figure 1A) and found that FTY720 and KRP203 decreased secretion of both Aβ40 and Aβ42 in a dose dependent manner (Fig. 1B). To further investigate the effect of these compounds, we tested a mouse neuronal N2a cell line stably expressing GFP, truncated Notch fused with Gal4/VP16 (NΔE/gvp) and a luciferase reporter under UAS promoter [32], [33], [36] (N2aNH cell line), which enables us to simultaneously analyze the effects of compounds on Aβ production, Notch signaling (as luciferase activity) and cell viability (as GFP fluorescence). Treatment with FTY720 decreased the production of Aβ40 and Aβ42 in a dose dependent fashion below the toxic concentration (Fig. 1C and S1) without affecting the Notch signaling (Fig. 1D and E).

**Figure 2:**
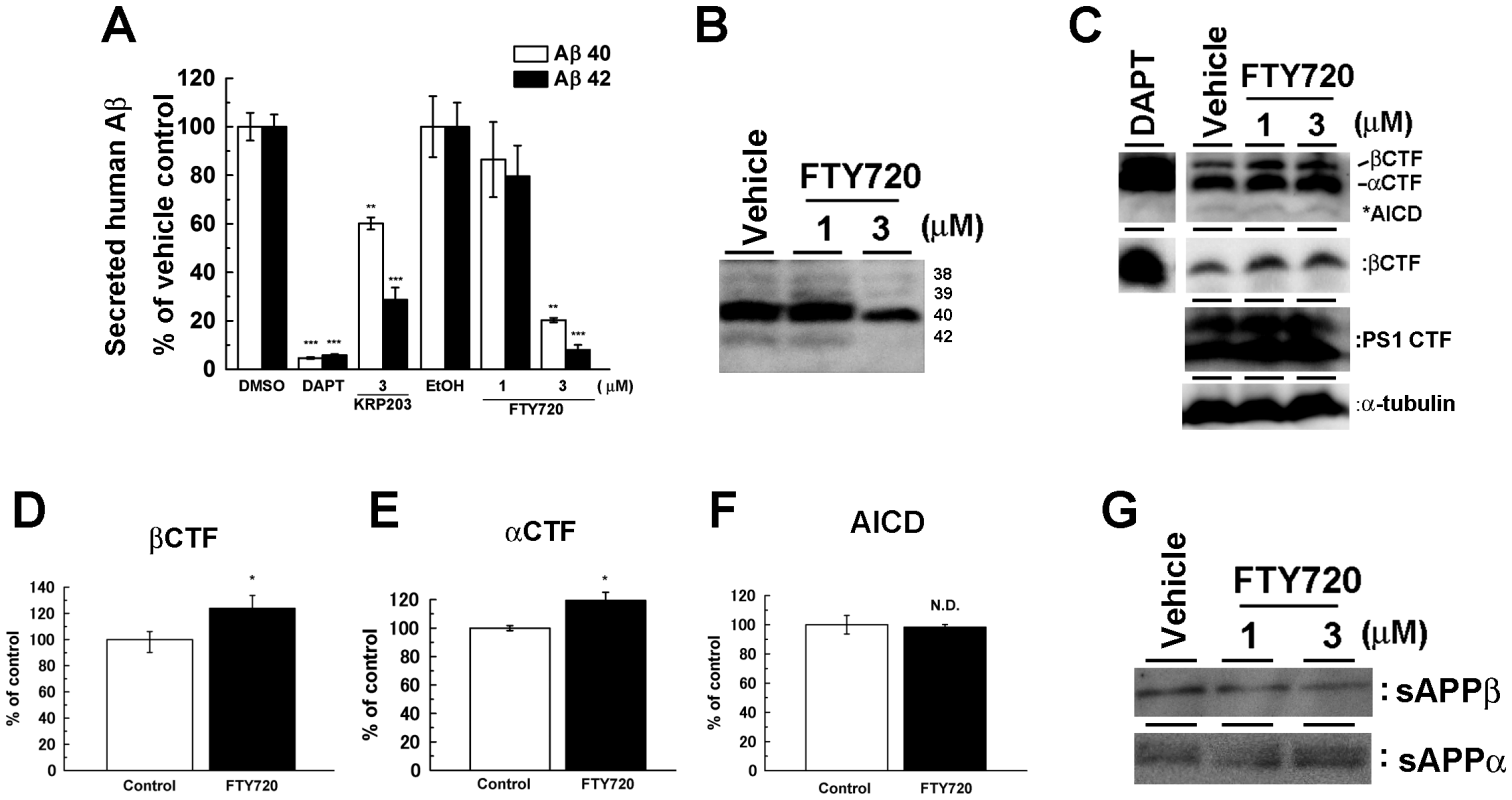
FTY720 decreased the γ-secretase-mediated cleavage of APP. SC100 were transiently transfected in N2a cells. After 24 hrs transfection, cells were treated with FTY720 or KRP203 for 24 hrs. (**A**) Levels of secreted human Aβ detected by human Aβ-specific ELISA (n = 4, mean ± SEM **P<0.01, ***P<0.001). (**B**) Immunoblotting analysis of secreted human Aβ separated by modified Tris/Tricin/8M Urea gel system. (**C**) Immunoblot analysis of APP CTFs including overexpressed SC100 and endogenous PS1 in FTY720-treated cell lysates. Quantification analysis of (**C**) for βCTF (**D**), αCTF (**E**) and AICD (**F**) (n = 4, mean ± SEM *P<0.05). (**G**) Immunoblot analysis of endogenous sAPPα and sAPPβ in the conditioned media of N2a cells.

To further identify the molecular target of S1PR modulators, we analyzed their effect on N2a cells transiently expressing SC100, corresponding to βCTF of human APP that is a direct substrate of γ-secretase. SC100 is endoproteolyzed by γ-secretase at ε-site to release the intracellular domain (AICD), and resultant intramembrane stub is trimmed by carboxypeptidase-like activity of the γ-secretase at multiple γ-sites to generate Aβ. FTY720 and KRP203 decreased secretion of Aβ40 and Aβ42 from SC100 (Fig. 2A), suggesting that S1PR modulators affected the γ-secretase activity. Recently, small compounds that specifically lower Aβ42, and Aβ40 to a lesser extent, have been termed as γ-secretase modulators [6], [29]. However, FTY720 treatment decreased all Aβ species with different Aβ C-termini, including Aβ38, Aβ39, Aβ40 and Aβ42 (Fig. 2B). Concomitantly, a slight accumulation of αCTF (from endogenous mouse APP) and SC100 was observed (Fig. 2C–F), whereas the levels of sAPPα and sAPPβ products of α- or β-secretase-mediated APP cleavage of endogenous APP, respectively, were not altered by FTY720 treatment (Fig. 2G). Intriguingly, AICD production was not altered by FTY720 treatment (Fig. 2F) similarly to the processing of NΔE, a direct Notch substrate for γ-secretase (see Fig. 1D and E). Because both AICD and NICD are produced from ε-cleavage by γ-secretase, these data suggest that S1PR modulators specifically regulate the γ-cleavage irrespective of the substrate.

### Mode of action of inhibition of Aβ production mediated by FTY720

**Figure 3:**
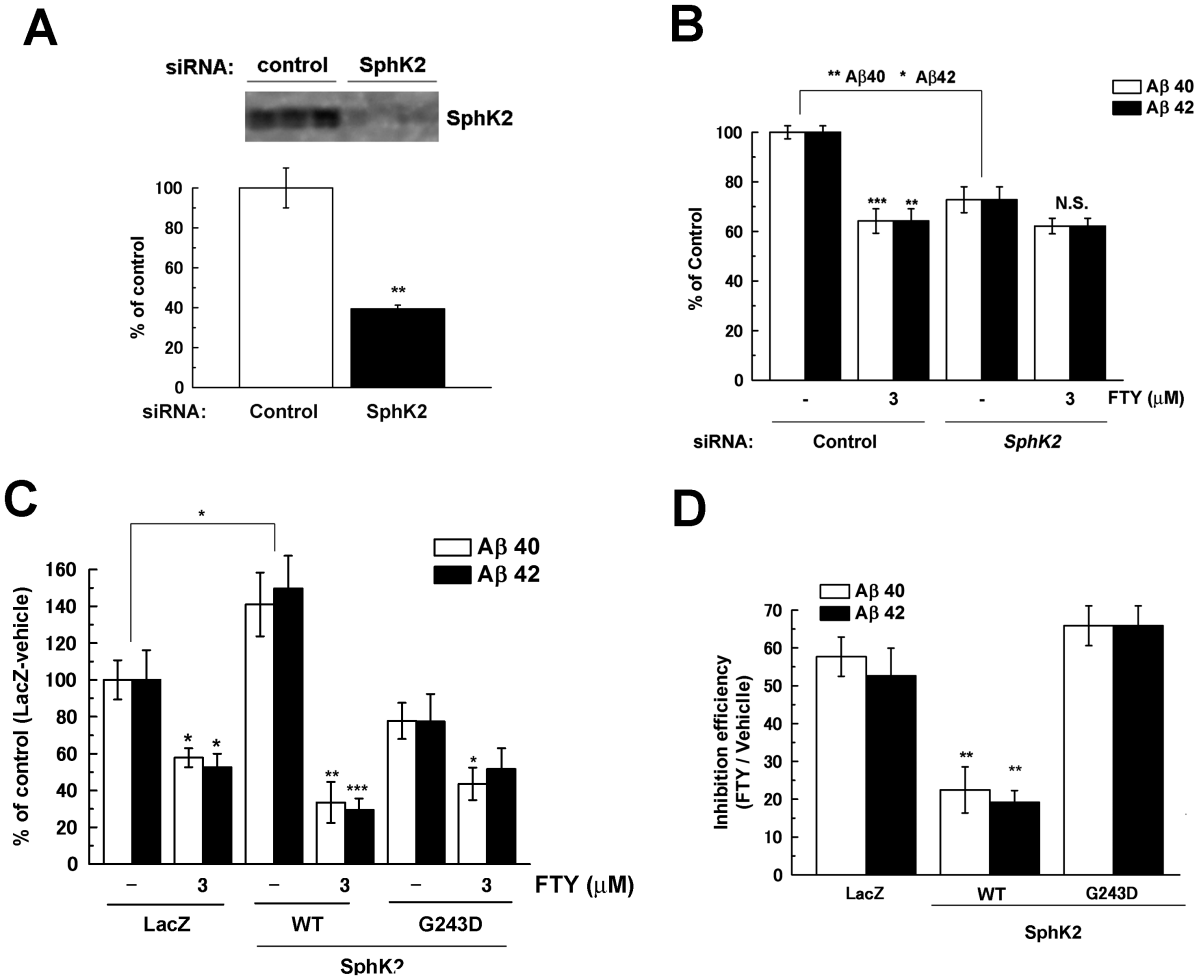
SphK2 activity is required for FTY720 mediated decrease of Aβ secretion. (**A**) N2a cells were transfected with siRNA against murine *SphK2*. After 48 hrs transfection of siRNA, levels of SphK2 was detected by immunoblotting (upper panel) and quantified (lower graph n = 3, mean ± SEM). (**B**) After 48 hrs transfection of siRNA, cells were treated with FTY720 for 24 hrs. Levels of secreted Aβ were quantified by ELISA (n = 3, mean ± SEM; *P<0.05, **P<0.01, ***P<0.001 compared with DMSO treatment or siRNA against *SphK2* (indicated by line)). One-way ANOVA with Tukey's post hoc test for individual treatment differences was used for statistical analysis. (**C**, **D**) N2a cells were transiently transfected with LacZ, wild-type (WT) or dominant negative mutant (G243D) SphK2. After 24 hrs transfection, cells were treated with FTY720 for 24 hrs. (**C**) Levels of secreted Aβ (n = 4, mean ± SEM; *P<0.05, **P<0.01, ***P<0.001 compared with DMSO treatment or SphK2 (indicated by line)). (**D**) The inhibitory efficiency of FTY720 on Aβ secretion compared with DMSO treatment in each transfection of (**C**). Secreted Aβ levels of FTY720 were standardized by vehicle control in each group (mean ± SEM; **P<0.01).

Phosphorylation of FTY720 by SphK2 yields the active metabolite, FTY720-phosphate (FTY720-P), which is known as a potent agonist of the S1P receptors (Fig. 1A). To determine whether FTY720-P is involved in the regulation of Aβ production, we treated N2a cells with FTY720 after RNAi knock-down of the endogenous expression of SphK2. We observed a ∼60% of decrease in SphK2 expression after siRNA treatment (Fig 3A). As reported previously, knockdown of SphK2 decreased Aβ secretion [22]. However, additional decrease was not observed in FTY720-treated SphK2 knockdown cells, suggesting that SphK2 is required for lowering Aβ secretion by FTY720 (Fig. 3B). Next, we examined the effects of overexpression of SphK2 or its dominant negative mutant (G243D) in N2a cells. As reported previously, overexpression of SphK2 increased Aβ production [22] (Fig. 3C). Intriguingly, FTY720 treatment significantly decreased Aβ secretion from N2a cells that overexpress wild-type (WT) SphK2, but not dominant negative mutant, to levels lower than those of untransfected cells treated by FTY720. Quantitative comparison of the inhibitory effects of FTY720 revealed that an increase in SphK2 activity significantly sensitized N2a cells to the inhibitory effect of FTY720 on Aβ production (Fig. 3D). These data strongly suggest SphK2 activity is involved in the mechanism of action of FTY720 to lower Aβ production, raising the possibility that FTY720-P is the *bona fide* regulator of the γ-secretase activities.

**Figure 4:**
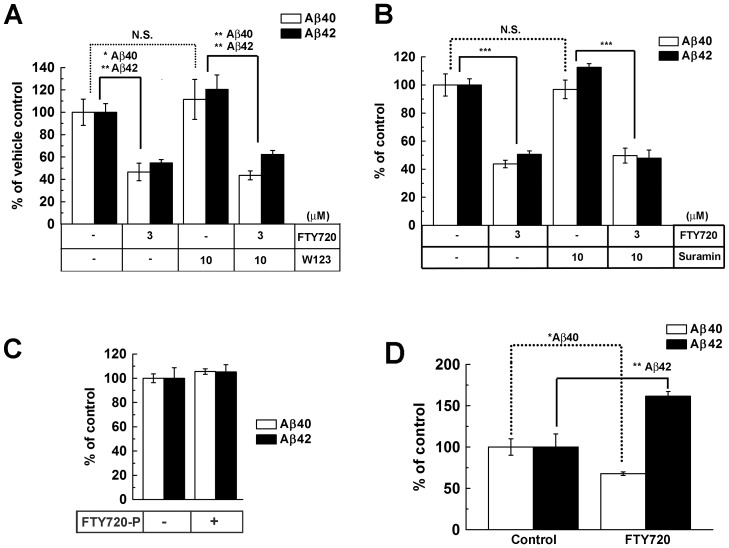
The effect of FTY720 on Aβ production is independent of downstream signaling of S1P receptors. (**A**) Levels of secreted Aβ from N2a cells co-treated with FTY720 and S1PR1 receptor antagonist W123 for 24 hrs (n = 4, mean ± SEM; *P<0.05, **P<0.01, N.S. no significant difference). (**B**) Levels of secreted Aβ from N2a cells co-treated with FTY720 and Gi protein inhibitor suramin for 24 hrs (n = 4, mean ± SEM; ***P<0.001, N.S. no significant difference). (**C**) Levels of secreted Aβ from N2a cells treated with FTY720-P for 24 hrs (n = 4, mean ± SEM). (**D**) *In vivo* effect of FTY720 on Aβ levels in AD model mice brain. Levels of soluble Aβ in the cerebral cortices of female A7 mice at 6 months of age after 6-days treatment with FTY720 (0.5 mg/kg/day, s.c.). Total brain human Aβ levels were measured by human-Aβ specific sandwich ELISA (n = 3–4, mean ± SEM, *P<0.05, ** p<0.01).

Phosphorylation of FTY720 by SphK takes place in the cytosol, and the resultant FTY720-P translocates to the extracellular side and acts as an agonist for S1PRs [38]. Next we examined whether known downstream signaling pathway of S1PRs was involved in the modulation of Aβ production. S1PR1, a major target of FTY720-P, is a Gi coupled receptor [39]. FTY720-P caused a significant phosphorylation of ERK1/2, a known downstream event of S1PR1-Gi signaling cascade (Fig. S2) in a similar fashion to that by SEW2871 [40]. Phosphorylation of ERK1/2 induced by SEW2871 was decreased by an authentic S1PR1 antagonist, W123 [41]. However, neither SEW2871 nor W123 affected the Aβ productions at indicated doses (Fig. S3). Then we tested co-treatment of W123 or suramin, the latter being known to work as a Gi protein inhibitor [42] together with FTY720. We found that both compounds failed to affect the decremental effect of FTY720 on Aβ production (Fig. 4A and B). In sharp contrast, extracellular addition of FTY720-P did not affect the Aβ production from N2a cells (Fig. 4C). These results raise the possibility that the molecular mechanism whereby FTY720 lowers Aβ production is independent of its antagonistic effects neither on S1PR1 nor Gi pathways and that intracellular FTY720-P lowers Aβ by an as yet identified mechanism.

### *In vivo* effects of FTY720 on Aβ levels in brains of AD model mice

FTY720 is known to cross the blood brain barrier and be accumulated in brains to suppress the inflammatory response in model mice of MS [19]. These data prompted us to test the *in vivo* effects of FTY720 in AD model mice. We subcutaneously injected FTY720 (0.5 mg/kg/day) for 6 days into 6-month-old female A7 transgenic mice overexpressing human APP carrying Swedish and Austrian double mutations [37]. At 6 months, A7 mice do not show amyloid plaques, whereas the levels of soluble Aβ are moderately increased compared to those in younger animals. Unexpectedly, we found that the level of soluble Aβ40 was decreased, whereas that of Aβ42 was significantly increased by a 6-days treatment with FTY720. This suggests that FTY720 treatment impacts on brain Aβ levels *in vivo*, although with some difference to its cellular effects.

## Discussion

In this study, we examined the effect of S1PR modulators, FTY720 and KRP203, on brain Aβ and showed these compounds decreased Aβ production in neuronal cells. S1PR modulators decreased Aβ production also from βCTF, an APP-derived direct substrate of γ-secretase, without affecting AICD production nor the cleavage of Notch, suggesting that these reagents inhibited the carboxypeptidase-like γ-secretase activity. Sphk2 activity was required for the FTY720-mediated decrease of Aβ production, whereas the signaling cascade downstream of S1PR was dispensable for the effects of the FTY720. FTY720 was active also *in vivo* and decreased the levels of Aβ40 but increased those of Aβ42 in brains of APP transgenic mice. These results shed light on the complex regulatory function of S1PR modulators on brain Aβ.

Phosphorylation of FTY720 or KRP203 by SphK2 yields the active metabolite FTY720-P or KRP203-P, respectively, which acts as the ligand for S1PR1 [14], [15], [38]. It has been shown that phosphorylated S1PR modulators induce endocytosis of S1PR1 from the cell surface after binding to S1PR1, thereby causing the antagonistic effects [14], [15]. Our RNAi suppression and overexpression studies showed that the SphK2 level was correlated to the capacity of FTY720 to decrease Aβ production (Fig. 3), supporting the view that phosphorylated forms of S1PR modulators are the *bona fide* active species that lower Aβ production. However, neither an authentic S1PR1 receptor an agonist SEW2871 nor antagonist W123 altered the Aβ production (Fig. S3). Moreover, co-treatment with W123 [41] or suramin [42] failed to cancel the inhibitory effect of FTY720 (Fig. 4), raising the possibility that the unconventional GPCR cascade is involved in the modulation of Aβ production. Interestingly, it has been shown that β2 adrenergic receptor [43] and an orphan G-protein receptor GPR3, the latter exhibiting a significant homology to S1PRs [44], affect the γ-secretase activity through the β-arrestin pathway [45], [46], [47]. β-Arrestins redistributes the γ-secretase complex toward detergent-resistant membranes, thereby increasing the catalytic activity of the complex [45], [47]. This raises the possibility that S1PR modulators also alter the distribution of γ-secretase to regulate the APP-specific processing to generate Aβ. Another possibility would be that FTY720-P directly binds to γ-secretase or APP. FTY720 is known to accumulate within endosomes [48], in which mature γ-secretase and APP reside [10]. Interestingly, it has been shown that phosphatidylinositol 4,5-diphosphate (PI(4,5)P2), a representative phospholipid with a second messenger activity, directly binds to γ-secretase and inhibits its activity [49]. However, we observed extracellularly treated FTY720-P did not significantly alter the Aβ production. It would be important to note that controlled phosphorylation of S1PR modulators at appropriate intracellular locations is critical to their function [50]. Moreover, we are unable to rule out the possibility that other S1PRs or unknown receptor is involved in the FTY720-mediated γ-secretase inhibition. Further study is required to determine the precise intracellular site of phosphorylation, and distribution, of FTY720-P in specific membrane microdomains or organelles. The binding of FTY720-P to γ-secretase or APP should also be examined.

In contrast to the *in vitro* results in cells, a 6-days treatment of FTY720 decreased Aβ40, but increased Aβ42 in the brains of APP transgenic mice *in vivo* (Fig. 4D). We used a similar dosage of FTY720 that had been adopted in autoimmune model mice, which is expected to yield submicromolar levels of FTY720 in brains [19]. Thus, the unexpected rise in Aβ42 levels caused by FTY720 *in vivo* in brains might have been due to other mechanisms that are distinct from the modulation of the γ-secretase activity in cultured cells. Another possibility is that FTY720 affected the Aβ42 levels by negatively regulating the inflammatory responses in the central nervous system. FTY720 has been reported to inhibit the egress of T cell into the spinal cord in autoinflammatory response [19], as well as into the ischemic lesions in brain ischemia [51]. Interestingly, FTY720 also inhibited the migration of human monocytes induced by Aβ42 [52]. It is tempting to speculate that the FTY720 might have impacted on the Aβ42 metabolism *in vivo* through altering the clearance of Aβ42 by microglial cells, overriding the inhibitory effects on neurons. As FTY720 has been approved for MS therapy in clinics, the regulatory mechanisms whereby S1PR modulators impact on Aβ42 metabolism, as well as on the inflammatory responses in AD brains, should further be characterized, in the light of therapeutics as well as adverse effects in humans. In sum, we identified a novel role of S1PR modulators on Aβ, which may open up a novel aspect in Aβ metabolism and lead to a novel therapeutic strategy for AD.

## Supporting Information

Figure S1 **AlamarBlue assay of N2aNH cells treated with S1PR modulators.** After 24 hr treatment with S1PR modulators, N2aNH cells were incubated with cultured medium containing almarBlue (Invitrogen). Medium was collected to monitor fluorescence at 530–560/590 nm excitation/emission wavelengthes.
(TIF)

Figure S2 **Effect of S1PR1 agonist and antagonist on ERK1/2 phosphorylation.** Immunoblotting analysis of N2a cell lysates for ERK1/2 phosphorylation. Prior to stimulation with the indicated compounds, N2a cells were starved in serum free medium for 6 hr. Results of densitometric analysis of phosphorylated ERK1/2 (compared with control) are shown below the columns. (**A**) N2a cells were incubated for 10 min with the FTY720 (1 µM), FTY720-P (1 µM), SEW2871 (1 µM), and PKC activator PMA (Phorbol 12-Myristate 13-acetate; 1 µM), which in known as an activator of ERK1/2 phosphorylation. (**B**) N2a cells were preincubated with or without 1 µM W123 for 10 min. After addition of 1 µM SEW2871, cells were further incubated for 30 min and harvested for immunoblotting.
(TIF)

Figure S3 **Dose dependent responses of S1PR1 agonist and antagonist on N2aNH cells.** N2aNH cells were treated with SEW2871 or W123 for 24 hr at indicated doses. These reagents have no toxicity at 0.3, 1, 3, and 10 µM (**A**; alamarBlue assay), and failed to affect the Aβ40 production at indicated doses (**B**).
(TIF)

## References

[1] Haughey NJ, Bandaru VV, Bae M, Mattson MP (2010) Roles for dysfunctional sphingolipid metabolism in Alzheimer's disease neuropathogenesis. Biochimica et biophysica acta 1801: 878–886. 20452460 10.1016/j.bbalip.2010.05.003 PMC2907186

[2] Cutler RG, Kelly J, Storie K, Pedersen WA, Tammara A, et al (2004) Involvement of oxidative stress-induced abnormalities in ceramide and cholesterol metabolism in brain aging and Alzheimer's disease. Proceedings of the National Academy of Sciences of the United States of America 101: 2070–2075. 14970312 10.1073/pnas.0305799101 PMC357053

[3] Han X, D MH, McKeel DW Jr, Kelley J, Morris JC (2002) Substantial sulfatide deficiency and ceramide elevation in very early Alzheimer's disease: potential role in disease pathogenesis. Journal of neurochemistry 82: 809–818. 12358786 10.1046/j.1471-4159.2002.00997.x

[4] He X, Huang Y, Li B, Gong CX, Schuchman EH (2010) Deregulation of sphingolipid metabolism in Alzheimer's disease. Neurobiology of aging 31: 398–408. 18547682 10.1016/j.neurobiolaging.2008.05.010 PMC2829762

[5] Grimm MO, Rothhaar TL, Hartmann T (2012) The role of APP proteolytic processing in lipid metabolism. Experimental brain research Experimentelle Hirnforschung Experimentation cerebrale 217: 365–375. 22179528 10.1007/s00221-011-2975-6

[6] Tomita T (2009) Secretase inhibitors and modulators for Alzheimer's disease treatment. Expert review of neurotherapeutics 9: 661–679. 19402777 10.1586/ern.09.24

[7] De Strooper B, Vassar R, Golde T (2010) The secretases: enzymes with therapeutic potential in Alzheimer disease. Nature reviews Neurology 6: 99–107. 20139999 10.1038/nrneurol.2009.218 PMC2879045

[8] Vassar R, Kovacs DM, Yan R, Wong PC (2009) The beta-secretase enzyme BACE in health and Alzheimer's disease: regulation, cell biology, function, and therapeutic potential. The Journal of neuroscience: the official journal of the Society for Neuroscience 29: 12787–12794. 19828790 10.1523/JNEUROSCI.3657-09.2009 PMC2879048

[9] Takasugi N, Tomita T, Hayashi I, Tsuruoka M, Niimura M, et al (2003) The role of presenilin cofactors in the gamma-secretase complex. Nature 422: 438–441. 12660785 10.1038/nature01506

[10] Vetrivel KS, Thinakaran G (2010) Membrane rafts in Alzheimer's disease beta-amyloid production. Biochimica et biophysica acta 1801: 860–867. 20303415 10.1016/j.bbalip.2010.03.007 PMC2886169

[11] Kalvodova L, Kahya N, Schwille P, Ehehalt R, Verkade P, et al (2005) Lipids as modulators of proteolytic activity of BACE: involvement of cholesterol, glycosphingolipids, and anionic phospholipids in vitro. The Journal of biological chemistry 280: 36815–36823. 16115865 10.1074/jbc.M504484200

[12] Osenkowski P, Ye W, Wang R, Wolfe MS, Selkoe DJ (2008) Direct and potent regulation of gamma-secretase by its lipid microenvironment. The Journal of biological chemistry 283: 22529–22540. 18539594 10.1074/jbc.M801925200 PMC2504869

[13] Holmes O, Paturi S, Ye W, Wolfe MS, Selkoe DJ (2012) Effects of membrane lipids on the activity and processivity of purified gamma-secretase. Biochemistry 51: 3565–3575. 22489600 10.1021/bi300303g PMC3347702

[14] Brinkmann V, Billich A, Baumruker T, Heining P, Schmouder R, et al (2010) Fingolimod (FTY720): discovery and development of an oral drug to treat multiple sclerosis. Nat Rev Drug Discov 9: 883–897. 21031003 10.1038/nrd3248

[15] Aktas O, Kury P, Kieseier B, Hartung HP (2010) Fingolimod is a potential novel therapy for multiple sclerosis. Nat Rev Neurol 6: 373–382. 20551946 10.1038/nrneurol.2010.76

[16] Zemann B, Kinzel B, Muller M, Reuschel R, Mechtcheriakova D, et al (2006) Sphingosine kinase type 2 is essential for lymphopenia induced by the immunomodulatory drug FTY720. Blood 107: 1454–1458. 16223773 10.1182/blood-2005-07-2628

[17] Don AS, Martinez-Lamenca C, Webb WR, Proia RL, Roberts E, et al (2007) Essential requirement for sphingosine kinase 2 in a sphingolipid apoptosis pathway activated by FTY720 analogues. J Biol Chem 282: 15833–15842. 17400555 10.1074/jbc.M609124200

[18] Hogenauer K, Billich A, Pally C, Streiff M, Wagner T, et al (2008) Phosphorylation by sphingosine kinase 2 is essential for in vivo potency of FTY720 analogues. ChemMedChem 3: 1027–1029. 18383466 10.1002/cmdc.200800037

[19] Foster CA, Howard LM, Schweitzer A, Persohn E, Hiestand PC, et al (2007) Brain penetration of the oral immunomodulatory drug FTY720 and its phosphorylation in the central nervous system during experimental autoimmune encephalomyelitis: consequences for mode of action in multiple sclerosis. J Pharmacol Exp Ther 323: 469–475. 17682127 10.1124/jpet.107.127183

[20] Choi JW, Gardell SE, Herr DR, Rivera R, Lee CW, et al (2011) FTY720 (fingolimod) efficacy in an animal model of multiple sclerosis requires astrocyte sphingosine 1-phosphate receptor 1 (S1P1) modulation. Proceedings of the National Academy of Sciences of the United States of America 108: 751–756. 21177428 10.1073/pnas.1014154108 PMC3021041

[21] Fujino M, Funeshima N, Kitazawa Y, Kimura H, Amemiya H, et al (2003) Amelioration of experimental autoimmune encephalomyelitis in Lewis rats by FTY720 treatment. The Journal of pharmacology and experimental therapeutics 305: 70–77. 12649354 10.1124/jpet.102.045658

[22] Takasugi N, Sasaki T, Suzuki K, Osawa S, Isshiki H, et al (2011) BACE1 Activity Is Modulated by Cell-Associated Sphingosine-1-Phosphate. The Journal of neuroscience: the official journal of the Society for Neuroscience 31: 6850–6857. 21543615 10.1523/JNEUROSCI.6467-10.2011 PMC4534000

[23] Kaneko T, Murakami T, Kawana H, Takahashi M, Yasue T, et al (2006) Sphingosine-1-phosphate receptor agonists suppress concanavalin A-induced hepatic injury in mice. Biochemical and biophysical research communications 345: 85–92. 16674913 10.1016/j.bbrc.2006.04.067

[24] Kan T, Tominari Y, Morohashi Y, Natsugari H, Tomita T, et al.. (2003) Solid-phase synthesis of photoaffinity probes: highly efficient incorporation of biotin-tag and cross-linking groups. Chemical communications: 2244–2245. 10.1039/b306970b 13678222

[25] Tomita T, Takikawa R, Koyama A, Morohashi Y, Takasugi N, et al (1999) C terminus of presenilin is required for overproduction of amyloidogenic Abeta42 through stabilization and endoproteolysis of presenilin. The Journal of neuroscience: the official journal of the Society for Neuroscience 19: 10627–10634. 10594046 10.1523/JNEUROSCI.19-24-10627.1999 PMC6784929

[26] Tomita T, Maruyama K, Saido TC, Kume H, Shinozaki K, et al (1997) The presenilin 2 mutation (N141I) linked to familial Alzheimer disease (Volga German families) increases the secretion of amyloid beta protein ending at the 42nd (or 43rd) residue. Proceedings of the National Academy of Sciences of the United States of America 94: 2025–2030. 9050898 10.1073/pnas.94.5.2025 PMC20036

[27] Iwatsubo T, Odaka A, Suzuki N, Mizusawa H, Nukina N, et al (1994) Visualization of A beta 42(43) and A beta 40 in senile plaques with end-specific A beta monoclonals: evidence that an initially deposited species is A beta 42(43). Neuron 13: 45–53. 8043280 10.1016/0896-6273(94)90458-8

[28] Qi-Takahara Y, Morishima-Kawashima M, Tanimura Y, Dolios G, Hirotani N, et al (2005) Longer forms of amyloid beta protein: implications for the mechanism of intramembrane cleavage by gamma-secretase. The Journal of neuroscience: the official journal of the Society for Neuroscience 25: 436–445. 15647487 10.1523/JNEUROSCI.1575-04.2005 PMC6725472

[29] Merhi A, Andre B (2012) Internal amino acids promote Gap1 permease ubiquitylation via TORC1/Npr1/14-3-3-dependent control of the Bul arrestin-like adaptors. Mol Cell Biol 32: 4510–4522. 22966204 10.1128/MCB.00463-12 PMC3486192

[30] Kopan R, Schroeter EH, Weintraub H, Nye JS (1996) Signal transduction by activated mNotch: importance of proteolytic processing and its regulation by the extracellular domain. Proceedings of the National Academy of Sciences of the United States of America 93: 1683–1688. 8643690 10.1073/pnas.93.4.1683 PMC40002

[31] Iwata H, Tomita T, Maruyama K, Iwatsubo T (2001) Subcellular compartment and molecular subdomain of beta-amyloid precursor protein relevant to the Abeta 42-promoting effects of Alzheimer mutant presenilin 2. The Journal of biological chemistry 276: 21678–21685. 11283011 10.1074/jbc.M007989200

[32] Imamura Y, Watanabe N, Umezawa N, Iwatsubo T, Kato N, et al (2009) Inhibition of gamma-secretase activity by helical beta-peptide foldamers. Journal of the American Chemical Society 131: 7353–7359. 19432477 10.1021/ja9001458

[33] Becuwe M, Herrador A, Haguenauer-Tsapis R, Vincent O, Leon S (2012) Ubiquitin-mediated regulation of endocytosis by proteins of the arrestin family. Biochem Res Int 2012: 242764. 22988512 10.1155/2012/242764 PMC3439951

[34] Fukumoto H, Tomita T, Matsunaga H, Ishibashi Y, Saido TC, et al (1999) Primary cultures of neuronal and non-neuronal rat brain cells secrete similar proportions of amyloid beta peptides ending at A beta40 and A beta42. Neuroreport 10: 2965–2969. 10549806 10.1097/00001756-199909290-00017

[35] Suzuki K, Hayashi Y, Nakahara S, Kumazaki H, Prox J, et al (2012) Activity-dependent proteolytic cleavage of neuroligin-1. Neuron 76: 410–422. 23083742 10.1016/j.neuron.2012.10.003

[36] Karlstrom H, Bergman A, Lendahl U, Naslund J, Lundkvist J (2002) A sensitive and quantitative assay for measuring cleavage of presenilin substrates. The Journal of biological chemistry 277: 6763–6766. 11744687 10.1074/jbc.C100649200

[37] Yamada K, Yabuki C, Seubert P, Schenk D, Hori Y, et al (2009) Abeta immunotherapy: intracerebral sequestration of Abeta by an anti-Abeta monoclonal antibody 266 with high affinity to soluble Abeta. The Journal of neuroscience: the official journal of the Society for Neuroscience 29: 11393–11398. 19741145 10.1523/JNEUROSCI.2021-09.2009 PMC6665926

[38] Takabe K, Paugh SW, Milstien S, Spiegel S (2008) "Inside-out" signaling of sphingosine-1-phosphate: therapeutic targets. Pharmacol Rev 60: 181–195. 18552276 10.1124/pr.107.07113 PMC2695666

[39] Lee MJ, Evans M, Hla T (1996) The inducible G protein-coupled receptor edg-1 signals via the G(i)/mitogen-activated protein kinase pathway. The Journal of biological chemistry 271: 11272–11279. 8626678 10.1074/jbc.271.19.11272

[40] Mullershausen F, Craveiro LM, Shin Y, Cortes-Cros M, Bassilana F, et al (2007) Phosphorylated FTY720 promotes astrocyte migration through sphingosine-1-phosphate receptors. Journal of neurochemistry 102: 1151–1161. 17488279 10.1111/j.1471-4159.2007.04629.x

[41] Wei SH, Rosen H, Matheu MP, Sanna MG, Wang SK, et al (2005) Sphingosine 1-phosphate type 1 receptor agonism inhibits transendothelial migration of medullary T cells to lymphatic sinuses. Nature immunology 6: 1228–1235. 16273098 10.1038/ni1269

[42] Miron VE, Jung CG, Kim HJ, Kennedy TE, Soliven B, et al (2008) FTY720 modulates human oligodendrocyte progenitor process extension and survival. Ann Neurol 63: 61–71. 17918267 10.1002/ana.21227

[43] Ni Y, Zhao X, Bao G, Zou L, Teng L, et al (2006) Activation of beta2-adrenergic receptor stimulates gamma-secretase activity and accelerates amyloid plaque formation. Nat Med 12: 1390–1396. 17115048 10.1038/nm1485

[44] Tanaka S, Ishii K, Kasai K, Yoon SO, Saeki Y (2007) Neural expression of G protein-coupled receptors GPR3, GPR6, and GPR12 up-regulates cyclic AMP levels and promotes neurite outgrowth. J Biol Chem 282: 10506–10515. 17284443 10.1074/jbc.M700911200

[45] Thathiah A, Spittaels K, Hoffmann M, Staes M, Cohen A, et al (2009) The orphan G protein-coupled receptor 3 modulates amyloid-beta peptide generation in neurons. Science 323: 946–951. 19213921 10.1126/science.1160649

[46] Liu X, Zhao X, Zeng X, Bossers K, Swaab DF, et al.. (2012) beta-Arrestin1 regulates gamma-secretase complex assembly and modulates amyloid-beta pathology. Cell Res. 10.1038/cr.2012.167 PMC3587707 23208420

[47] Thathiah A, Horre K, Snellinx A, Vandewyer E, Huang Y, et al.. (2012) beta-arrestin 2 regulates Abeta generation and gamma-secretase activity in Alzheimer's disease. Nat Med. 10.1038/nm.3023 23202293

[48] Lieven CJ, Ribich JD, Crowe ME, Levin LA (2012) Redox proteomic identification of visual arrestin dimerization in photoreceptor degeneration after photic injury. Invest Ophthalmol Vis Sci 53: 3990–3998. 22599583 10.1167/iovs.11-9321 PMC4453105

[49] Osawa S, Funamoto S, Nobuhara M, Wada-Kakuda S, Shimojo M, et al (2008) Phosphoinositides suppress gamma-secretase in both the detergent-soluble and -insoluble states. J Biol Chem 283: 19283–19292. 18480063 10.1074/jbc.M705954200

[50] Gomez-Raja J, Davis DA (2012) The beta-arrestin-like protein Rim8 is hyperphosphorylated and complexes with Rim21 and Rim101 to promote adaptation to neutral-alkaline pH. Eukaryot Cell 11: 683–693. 22427429 10.1128/EC.05211-11 PMC3346431

[51] Shichita T, Sugiyama Y, Ooboshi H, Sugimori H, Nakagawa R, et al (2009) Pivotal role of cerebral interleukin-17-producing gammadeltaT cells in the delayed phase of ischemic brain injury. Nature medicine 15: 946–950. 10.1038/nm.1999 19648929

[52] Kaneider NC, Lindner J, Feistritzer C, Sturn DH, Mosheimer BA, et al (2004) The immune modulator FTY720 targets sphingosine-kinase-dependent migration of human monocytes in response to amyloid beta-protein and its precursor. FASEB J 18: 1309–1311. 15208267 10.1096/fj.03-1050fje

