# Supplementary figures and images for: FTY720/Fingolimod, a Sphingosine Analogue, Reduces Amyloid-β Production in Neurons
Source: PLoS One. 2013 May 7;8(5):e64050. doi: 10.1371/journal.pone.0064050 (PMC3646787; doi:10.1371/journal.pone.0064050)

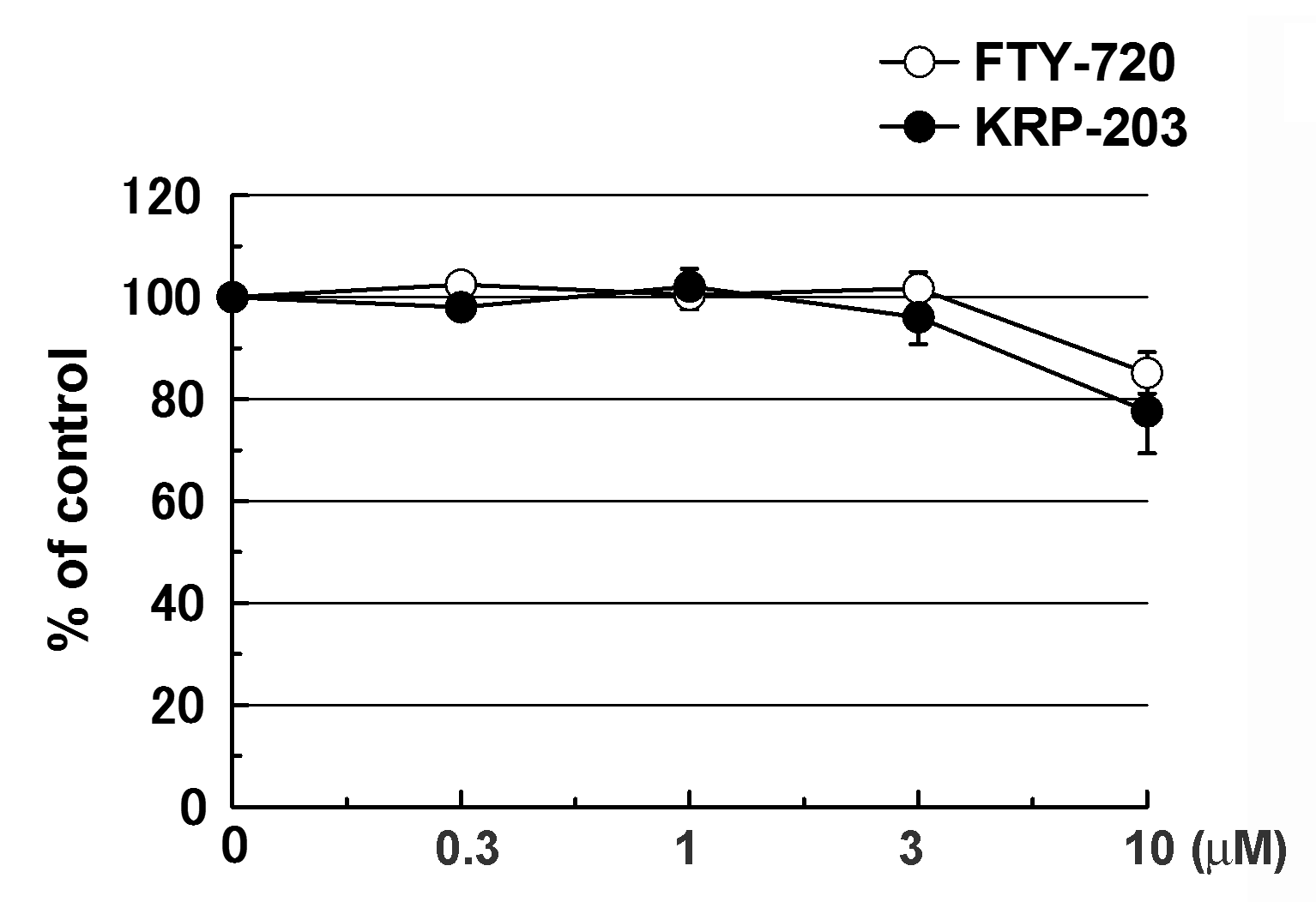

Supplement: Figure S1 — AlamarBlue assay of N2aNH cells treated with S1PR modulators. After 24 hr treatment with S1PR modulators, N2aNH cells were incubated with cultured medium containing almarBlue (Invitrogen). Medium was collected to monitor fluorescence at 530–560/590 nm excitation/emission wavelengthes. (TIF) [file pone.0064050.s001.tif]

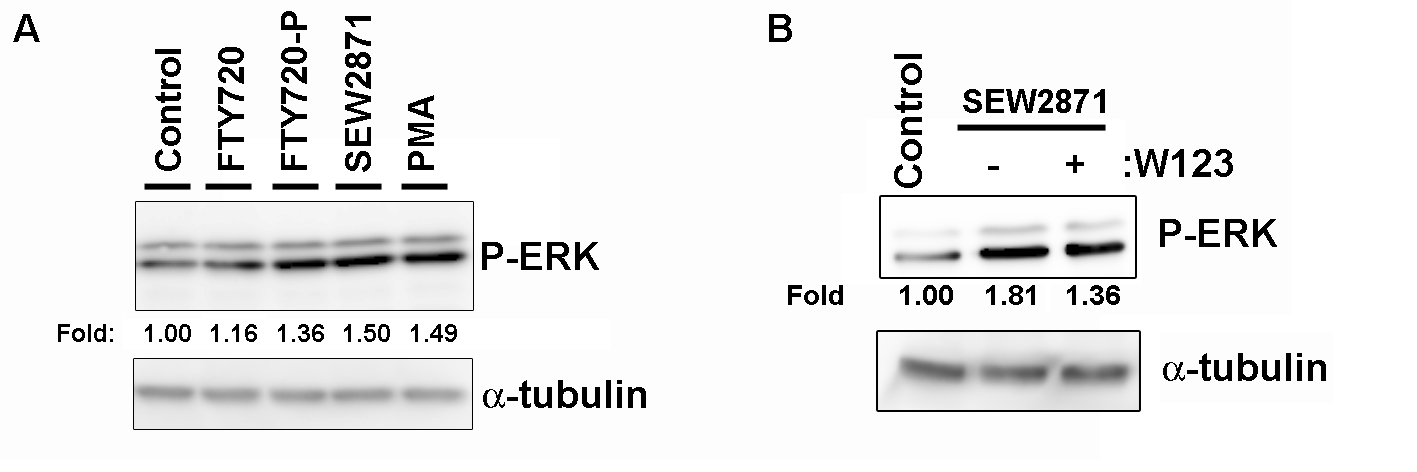

Supplement: Figure S2 — Effect of S1PR1 agonist and antagonist on ERK1/2 phosphorylation. Immunoblotting analysis of N2a cell lysates for ERK1/2 phosphorylation. Prior to stimulation with the indicated compounds, N2a cells were starved in serum free medium for 6 hr. Results of densitometric analysis of phosphorylated ERK1/2 (compared with control) are shown below the columns. (A) N2a cells were incubated for 10 min with the FTY720 (1 µM), FTY720-P (1 µM), SEW2871 (1 µM), and PKC activator PMA (Phorbol 12-Myristate 13-acetate; 1 µM), which in known as an activator of ERK1/2 phosphorylation. (B) N2a cells were preincubated with or without 1 µM W123 for 10 min. After addition of 1 µM SEW2871, cells were further incubated for 30 min and harvested for immunoblotting. (TIF) [file pone.0064050.s002.tif]

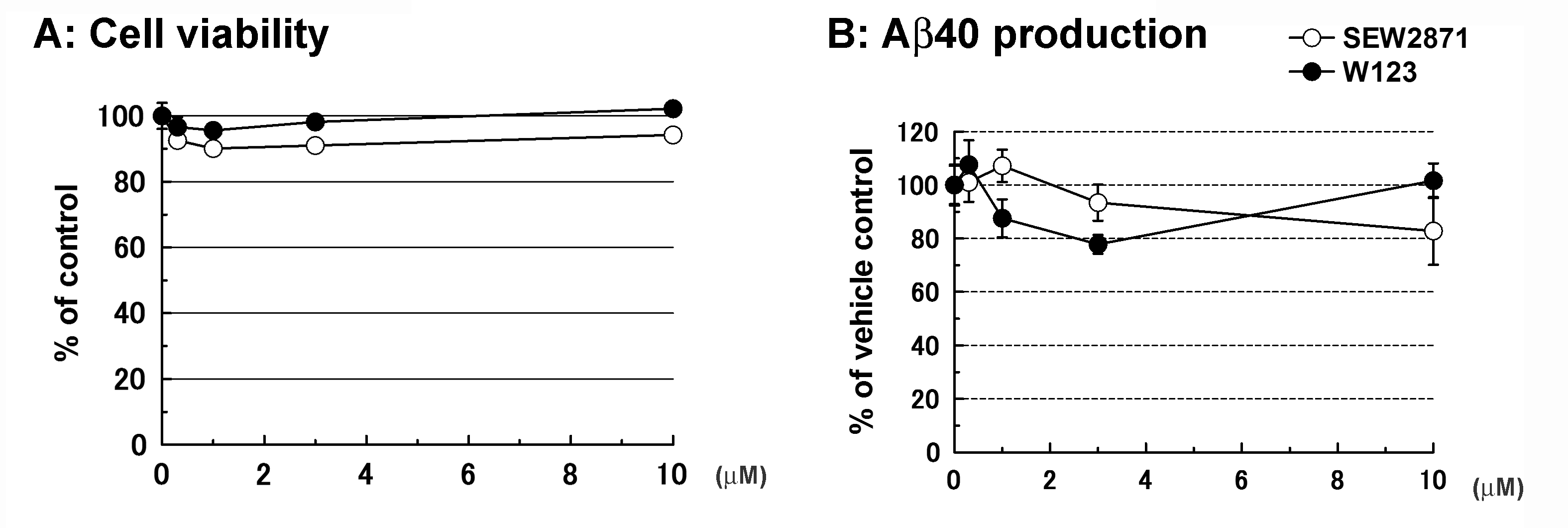

Supplement: Figure S3 — Dose dependent responses of S1PR1 agonist and antagonist on N2aNH cells. N2aNH cells were treated with SEW2871 or W123 for 24 hr at indicated doses. These reagents have no toxicity at 0.3, 1, 3, and 10 µM (A; alamarBlue assay), and failed to affect the Aβ40 production at indicated doses (B). (TIF) [file pone.0064050.s003.tif]
